# Reading Traits for Dynamically Presented Texts: Comparison of the Optimum Reading Rates of Dynamic Text Presentation and the Reading Rates of Static Text Presentation

**DOI:** 10.3389/fpsyg.2017.01390

**Published:** 2017-08-17

**Authors:** Miki Uetsuki, Junji Watanabe, Hideyuki Ando, Kazushi Maruya

**Affiliations:** ^1^Department of Contemporary Liberal Arts, Aoyama Gakuin Women’s Junior College Tokyo, Japan; ^2^Human Information Science Laboratory, Communication Science Laboratories, Nippon Telegraph and Telephone Corporation Kanagawa, Japan; ^3^Graduate School of Information Science and Technology, Osaka University Osaka, Japan

**Keywords:** dynamic text, static text, reading traits, impression of reading, silent reading, trace reading

## Abstract

With the growth in digital display technologies, dynamic text presentation is used widely in every day life, such as in electric advertisements and tickers on TV programs. Unlike static text reading, little is known about the basic characteristics underlying reading dynamically presented texts. Two experiments were performed to investigate this. Experiment 1 examined the optimum rate of dynamic text presentation in terms of a readability and favorability. This experiment demonstrated that, when the rate of text presentation was changed, there was an optimum presentation rate (around 6 letters/s in our condition) regardless of difficulty level. This indicates that the presentation rate of dynamic texts can affect the impression of reading. In Experiment 2, to elucidate the traits underlying dynamic text reading, we measured the reading speeds of silent and trace reading among the same participants and compared them with the optimum presentation rate obtained in Experiment 1. The results showed that the optimum rate was slower than with silent reading and faster than with trace reading, and, interestingly, the individual optimum rates of dynamic text presentation were correlated with the speeds of both silent and trace reading. In other words, the readers who preferred a fast rate in dynamic text presentation would also have a high reading speed for silent and trace reading.

## Introduction

Reading is an important cognitive process for acquiring and sharing information and has long been used with paper texts. In recent years, however, the opportunity to read texts displayed on a computer monitor has increased with the rapid development of digital media technologies (Zambarbieri and Carniglia, 2012; Margolin et al., 2013). Digital text presentation can be classified into two types in terms of temporal properties of the texts. In the first type, each letter is located at a position, and the luminance and colors of the letters are fixed [hereafter, we refer to this type as Static Text Presentation (STP)]—for example, the presentation of a book on a digital display. In STP, readers make a series of fixations and eye movements during reading (cf. Schotter et al., 2012) and can change the speed of reading voluntarily. In the second type, the position or luminance/color of the letters are temporally changed [we refer to this type as Dynamic Text Presentation (DTP)]—for example, scrolling texts on electric signboards, which are commonly seen in daily life. To read moving text on a signboard, readers are forced to track the texts with their eyes. Different types of DTP have been used in psychological experiments to examine the temporal characteristics of visual processing and memory in reading. For instance, in rapid serial visual presentation, sentences are presented word by word at a given rate and at a fixed position (Forster, 1970; Potter et al., 1980, 2008; Chen and Chien, 2007; Proaps and Bliss, 2014; Primativo et al., 2016). This was used to investigate temporal limits of sentence processing and memory in reading (Potter et al., 1980, 2008; Juola et al., 1982; Potter, 1984; Rubin and Turano, 1992; Benedetto et al., 2015) and to examine the process of syntactic structure by changing presentation rate (Forster, 1970; Forster and Ryder, 1971; Holmes and Forster, 1972; Chen, 1983; Schotter et al., 2014). Reading with moving windows (Just et al., 1982; Osaka and Osaka, 2002; Choi et al., 2015) is another type of DTP used in psychological experiments. A window moves from one word to another in a sentence, and the letters are visible only when the window is on them (otherwise, they are represented as dashes or dots) (Just et al., 1982). The letters are presented sequentially at different locations. In this way, DTP is used in daily life and as a tool to probe human reading processes in psychological experiments. However, the perceptual characteristics involved in reading DTP are not well-understood. Investigating the reading processes involved in reading DTP would contribute to designing a theory of DTP and would also elucidate underlying mechanisms of text reading. The present study examined the characteristics of reading dynamically presented texts while varying presentation parameters and compared the characteristics with those of other visual language processing.

**Key Concepts**

(1) Digital text presentation

Digital text presentation means that texts are presented digitally on displays of PC, TV, electronic book readers, or electronic scoreboards. It can be classified into two types. In the first type, each letter is fixed. In the second type, the position or luminance/color of letters are temporally changed.

(2) Static text presentation (STP)

In this presentation, the position or luminance/color of letters are fixed. For example, the presentation of a book on a digital display. Readers make a series of fixations and eye movements to read texts and can change the speed of reading voluntarily.

(3) Dynamic text presentation (DTP)

In this presentation, the position or luminance/color of letters are temporally changed. For example, scrolling texts on electronic signboards or TV. Dynamic text presentations have been used in psychological experiments to examine the temporal characteristics of reading, for example, RSVP and moving window methods. Readers are forced to track the texts with their eyes to read moving texts.

(4) An optimum presentation rate

An optimum presentation rate means that the speed that readers can read text easily or that readers favor when reading dynamic texts.

A primary parameter of DTP is the presentation rate. Digital display can present texts as both STP and DTP. However, while readers can control the speed of reading in STP, the reading speed cannot be controlled in DTP. As pointed out by Just et al. (1982) and Potter (1984), the perceptual processing of DTP might be similar to auditory language processing since the listeners must process speech at the speakers’ rate in auditory language as well. First, when the presentation rate is too fast, reading performance is degraded, because readers cannot understand the sentences (Potter, 1984; Miyake et al., 1994; Wang and Kan, 2004). This indicates that there should be a speed limit for the presentation rate of DTP. When the presentation rate of DTP is higher than this limit, readability will significantly decrease as the presentation rate increases. Conversely, the presentation rate limit will be observed as a point where readability significantly decreases. Next, when the presentation rate is too slow, it loses the rhythm in reading. In fact, under the minimal speed of reading, readers cannot extract information beyond individual words (Gibson and Levin, 1975, p. 539). The loss of rhythm in reading should lead to degradation in reading performance as in the case of reading with an excessively fast presentation rate. Additionally, when texts are presented at a slower speed, readers should remember the words for a longer time. The slower the speed of text presentation, the higher the memory load in reading, and the lower the reading performance. Considering these factors, there should be another limit in the presentation rate of DTP. When the presentation is slower than this limit, the reading performance will significantly decrease. Thus, both the excessively low and excessively high presentation rates result in decreased reading performance. If this hypothesis is accurate, the reading performance as a function of presentation rates will lead to an inverted U shape. This inverted U shape function gives an optimum presentation range in DTP.

In this paper, we examined this hypothesis in Experiment 1. In Experiment 2, the reading rates of silent (STP) and trace reading (active DTP) were measured to examine the relationship between the optimum rate and voluntary reading rates. This would show a possibility that the optimum DTP rate could be estimated easily by voluntary reading rates unless measuring the optimum DTP rates. Finally, we discuss the relevance of the optimum rate and the reading processes.

## Experiment 1

This experiment examined the temporal characteristics of reading dynamically presented texts while varying presentation parameters. Two independent variables were used in this experiment: presentation rate and text. Presentation rate is the primary parameter of DTP, and these texts were presented at nine rates that ranged widely to examine the optimum reading rate. The impression of DTP was focused in this experiment because some previous studies measured reading comprehension of DTP to prove reading or memory processes. Two text types of differing difficulties were used because text difficulty may affect a range of optimum rates. For example, it would be hard to comprehend a difficult text at high presentation rates because sentence processing of the text would take more time than that of an easy text. It would also hard to comprehend a difficult text at low presentation rates because memory load would be high. Thus, the sharpness of the inversed U shape functions of the texts would differ. Thus, two dependent variables, the rating value of favorability and readability, were used in this experiment. Readability was used because it was crucial for readers whether the presentation was easy to read. If the presentation that had high readability was not favored by readers, the presentation method was not desirable. Thus, favorability was also used.

This experiment examined the optimum presentation rate of DTP using a semantic differential method. If both the excessively low and excessively high presentation rates result in decreased reading performance, reading performance as a function of presentation rates will lead to an inversed U shape.

### Method

#### Participants

Nine junior college students and nine undergraduate and graduate students (11 males and 7 females; mean age 22.78 years, standard deviation [SD] 6.80) read the “Sunset” text, and nine junior college students and seven undergraduate and graduate students (9 males and 7 females; mean age 22.81 years, *SD*: 7.20) read the “Snow Country” text. Fifteen participants, including nine junior college students, read both texts. Written informed consent was obtained from all participants.

#### Stimuli

**FIGURE 1:**
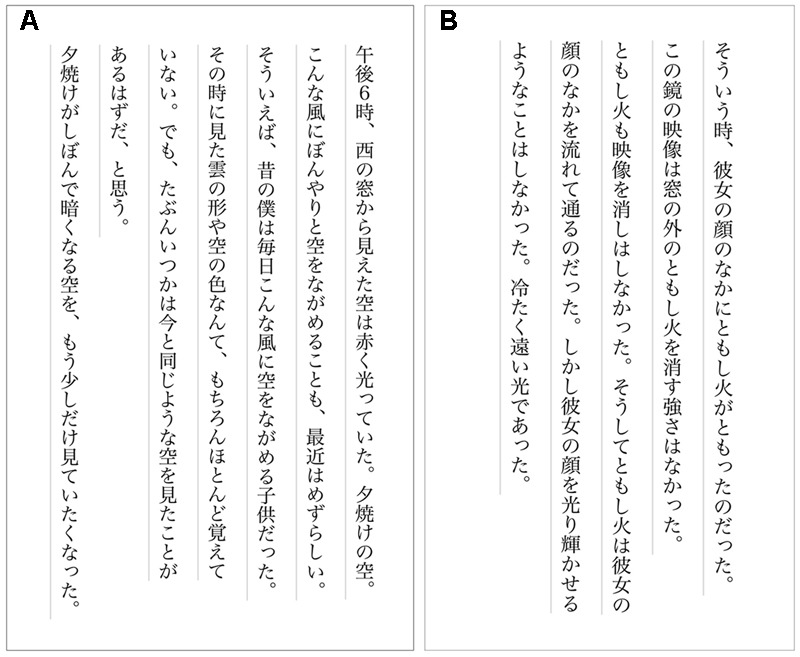
Stimulus texts used in this experiment **(A)** Sunset, **(B)** Snow Country.

Plain original text about a sunset and part of a dense novel called Snow Country by a distinguished Japanese writer, Kawabata (1947), were used as the stimuli (**Figures 1A,B**). Snow Country is more literary and should be more difficult than Sunset. The difficulty of the texts was examined by a questionnaire with twenty-five college students (25 female; mean age 19.00 years, *SD*: 1.51) that did not participate in this experiment. They judged the difficulty of the texts on a scale from 1 (very easy) to 7 (very difficult). The difficulty of Sunset was 2.16, and that of Snow Country was 4.76. Snow Country was significantly more difficult than Sunset (*t*[24] = 8.83, *p* < 0.0001). It was confirmed that Snow Country is difficult for most readers because it is more literary than Sunset. The letter size was about 5.5 mm, the space between lines was about 1 cm, and Hiragino Mincho Pro font was used. The letters were black, and the background was white, as shown in**Figure 1**.

Sunset: “I see the red sky from the western window at 6 p.m. The sunset sky. It is uncommon recently that I see the sky distractedly like now. I remember that I was a child that saw the sky like now every day. I hardly remember the shape of the clouds and the color of the sky that I saw in my childhood. But, I feel that I see the sky that I have seen. I come to want to see the darkening sky a little longer.” (183 letters, 204 morae in Japanese).

Snow Country: “It was then that a light shone in the face. The reflection in the mirror was not strong enough to blot out the light outside, nor was the light strong enough to dim the reflection. The light moved across the face, though not to light it up. It was a distant, cold light.” (135 letters, 154 morae in Japanese) (Kawabata, 1947/1957).

#### Procedure

**FIGURE 2:**
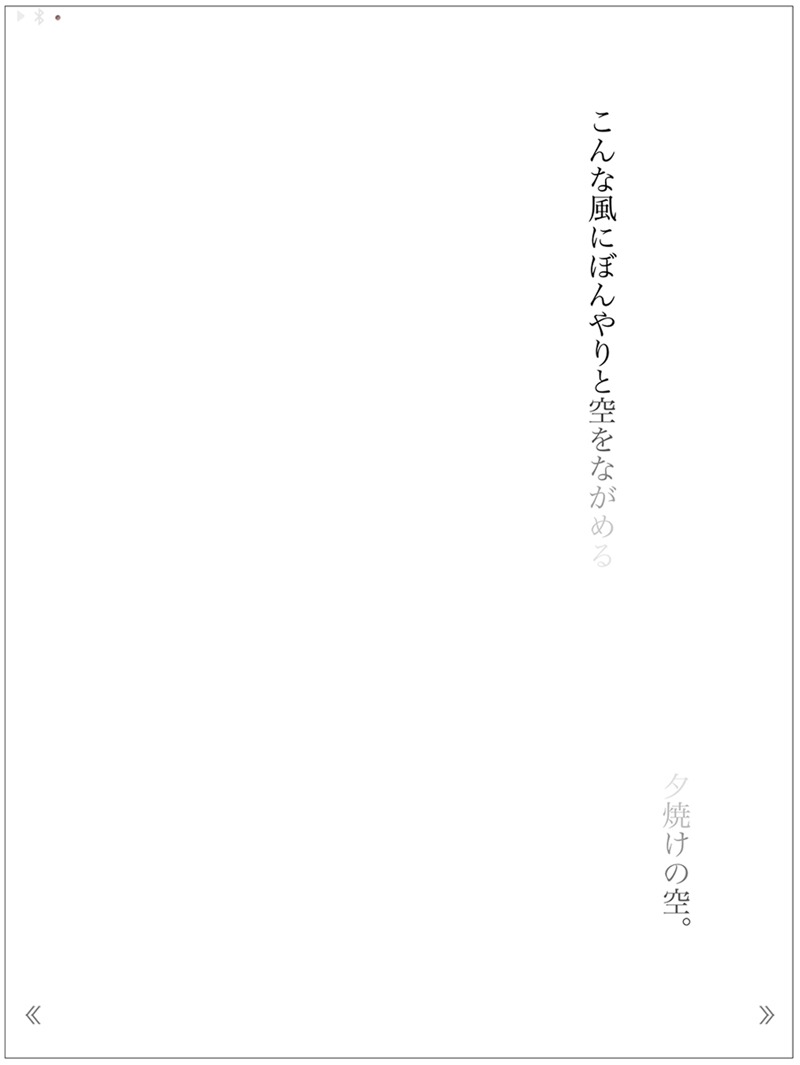
Example of the stimulus text presentation (Sunset, 6 letters/s).

We used a moving-window-like DTP in this experiment. The contrast of letters was barely visible at first, and, while the letters did not move, the contrast of the letters changed dynamically (**Figure 2**) (Maruya et al., 2012, 2013). The contrast of the letters gradually increased, stayed at the maximum level, and then decreased gradually to the initial level. The timing of contrast change for each letter was changed at a constant speed. Thus, the readers perceived that the letters appeared and disappeared as the contrast change moved at a constant speed. We chose a moving-window-like DTP because visual interferences like motion blur and visual masking exist in DTP. Motion blur is an apparent streaking of rapidly moving objects, which is caused by spatiotemporal integration of the visual system (Wong and Halvorsen, 2006), and DTP like the scroll presentation of letters causes severe motion blur. Visual masking is a phenomenon in which the visibility of a stimulus is degraded by another stimulus called a mask (Breitmeyer and Ganz, 1976; Breitmeyer and Ogmen, 2000), and it occurs in DTP as in the case of rapid serial visual presentation since the letters are presented at a rapid rate at the same location. In the moving-window-like DTP, neither motion blur nor visual masking occurs, and therefore, we can investigate traits underlying reading of dynamic texts without visual interference. In addition, a reader’s point of fixation and the reading position almost coincided with each other, and it was difficult to skip words and read regressively.

Texts were presented at nine rates that ranged from 2.4 to 12 letters/s (2.4, 2.7, 3, 3.5, 4, 5, 6, 8, 12 letters/s) using tablet computers (iPad). The contrast of the letters was barely visible at first. When a trial started, the contrast of the letters increased for 2 s, stayed at the maximum level for a second, and then decreased to the initial level for 2 s (**Figure 2**). The participants read the text silently, then made a judgment on its impression from -50 to 50 points using semantic differential scales on readability and favorability. The order of presentation rates was randomized. The same procedure was repeated three times for each participant (9 × 3 = 27 trials in total for each stimulus text), and the average points were used for the analysis.

### Results

**FIGURE 3:**
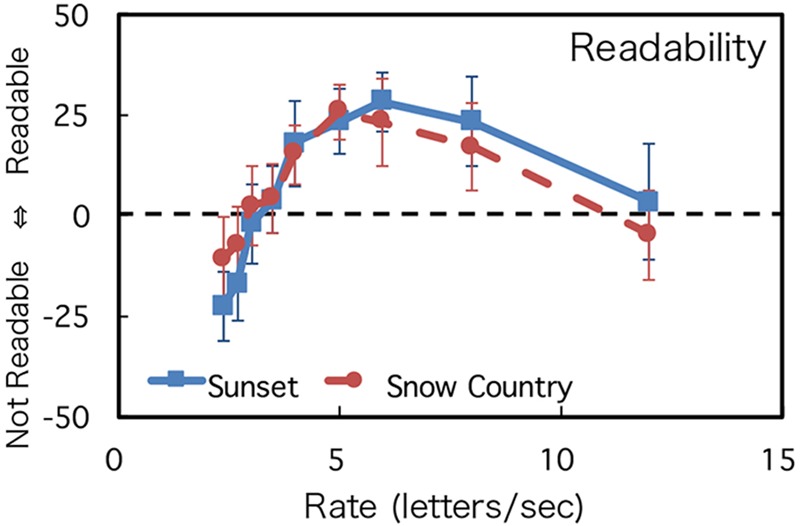
Rating of readability. Error bars show the 95% confidence intervals.

**FIGURE 4:**
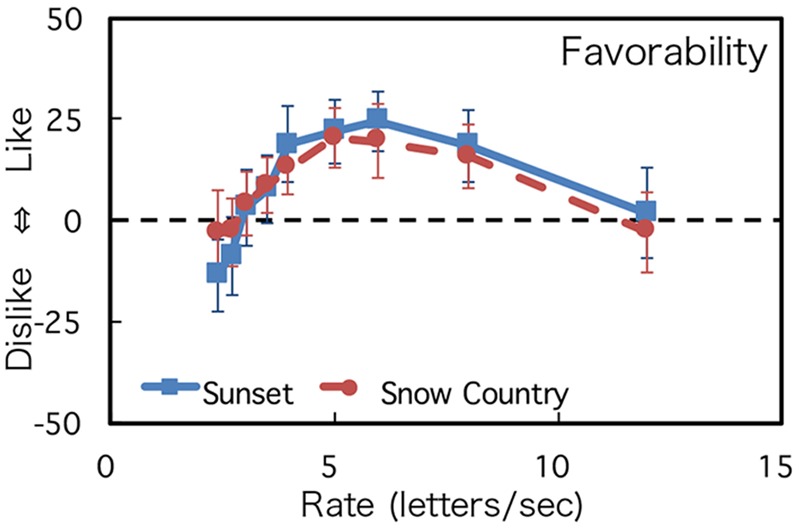
Rating of favorability. Error bars show the 95% confidence intervals.

The mean and SD of the participants’ ratings about readability and favorability are shown in **Figures 3, 4**. A two-way mixed analysis of variance (ANOVA) was conducted with the text type and presentation rate as factors and the rating value as the dependent variable for each judgment. The results of the ANOVA are shown in **Table 1**. The degrees of freedom were corrected using Greenhouse–Geisser correction as the Mauchly’s sphericity test was found to be significant.

**Table 1:** Results of the two-way mixed ANOVA of rating impression.

Impression	Effect	*F*-value, *p*-value, $\eta_{p}^{2}$-value	Multiple comparison (Bonferroni)
Readability	Rate	^∗∗∗^ *F*[2.93,93.6] = 26.83, *p* < 0.0001, $\eta_{p}^{2}$ = 0.46	12 letters/s < 5, 6, 8 letters/s, 2.4, 2.7, 3 letters/s < 8 letters/s, 2.4, 2.7, 3, 3.5 letters/s < 4, 5, 6 letters/s, 2.4, 2.7 letters/s < 3, 3.5 letters/s
	Text	*F*[1,32] = 0.04, n.s., $\eta_{p}^{2}$ = 0.00	–
	Rate × Text	*F*[2.93,93.6] = 1.34, n.s., $\eta_{p}^{2}$ = 0.04	–
Favorability	Rate	^∗∗∗^ *F*[3.17,102] = 17.12, *p* < 0.0001, $\eta_{p}^{2}$ = 0.35	12 letters/s < 5, 6, 8 letters/s, 3 letters/s < 4, 5, 6 letters/s, 3.5 letters/s < 5, 6 letters/s, 2.4, 2.7 letters/s < 3, 3.5, 4, 5, 6, 8 letters/s
	Text	*F*[1,32] = 0.01, n.s., $\eta_{p}^{2}$ = 0.00	–
	Rate × Text	*F*[3.17,102] = 0.92, n.s., $\eta_{p}^{2}$ = 0.03	–

*Degrees of freedom were corrected by Greenhouse–Geisser because Mauchly’s sphericity test was significant. ^∗∗∗^*p* < 0.0001.*

For both judgments, while the main effect of texts and the interaction between the texts and the rates were not significant, the main effect of presentation rate was significant. Rating values were low when presentation rates were slow, and, as the presentation rate increased, the rating values increased and reached the maximum level at the speed of around 6 letters/s. When the presentation rates increased, the rating values decreased again. These tendencies were observed in both readability and favorability. Experiment 1 demonstrated that the change of impression showed an inverted U shape and that there was an optimum DTP rate. However, the effect of text difficulties was not significant in ANOVA. This point will be discussed in the Section “Discussion.”

**FIGURE 5:**
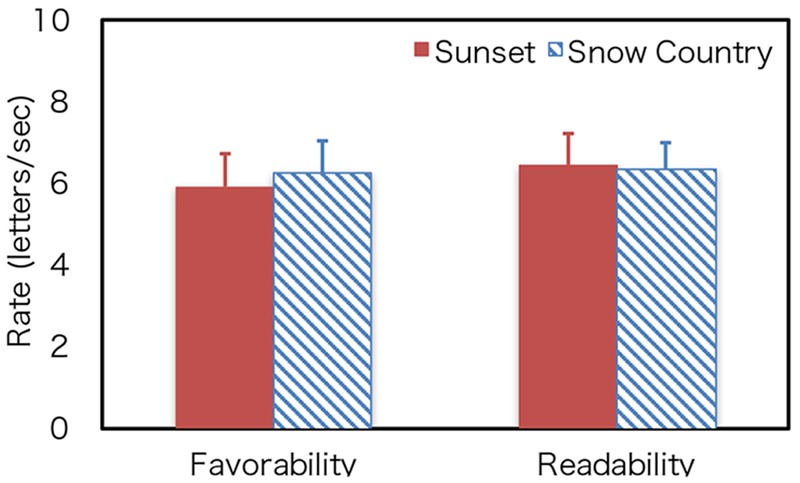
Optimum rate. Error bars show the 95% confidence intervals.

To quantitatively estimate the optimum speed of readability and favorability, each participant’s datasets were fitted to a log Gaussian function except for four individual datasets; three datasets (one for Sunset and two for Snow Country) did not converge, and one dataset (Sunset) was identified as an outlier through a Smirnov-Grubbs test. Thirty datasets were used for the analyses (16 for Sunset and 14 for Snow Country). The average coefficients of determination of the fitting were 0.84 (Sunset) and 0.77 (Snow Country) for readability and 0.76 (Sunset) and 0.70 (Snow Country) for favorability. **Figure 5** shows the average optimum rate estimated by the fitting. Readability for Sunset was 6.45 letters/s, and its favorability rate was 5.91 letters/s. Readability for Snow Country was 6.37 letters/s, and its favorability rate was 6.27 letters/s. There was no significant difference between the two texts for each judgment (Readability: *t*[28] = 0.81, n.s.; Favorability: *t*[28] = -0.03, n.s.). These results demonstrated that there are optimum rates for readability and favorability in our DTP, and the rates were almost the same for the two judgments. This indicates that the rates were independent of difficulty levels of the texts and were around 6 letters/s for our text stimuli.

## Experiment 2

In Experiment 2, to examine the relationship between the optimum DTP rate and voluntary reading rate, we measured rates of different visual reading processes, such as silent and trace reading, and compared them with the optimum rate obtained in Experiment 1. This would show the possibility that an optimum rate could be estimated easily by voluntary reading rates, unless measuring the optimum DTP rate. If this is the case, setting an appropriate DTP rate should be easy, because it requires additional time and effort to estimate the optimum DTP rate as shown in Experiment 1 than measuring voluntary reading rates.

While the reading of DTP in Experiment 1 was a passive reading process, silent and trace readings are voluntary reading processes. In silent reading, all letters were always presented at the maximum contrast (STP), and the reader read it in his/her mind. Silent reading would be fastest, since texts were read without voice production, and the readers could see the preceding letters. In trace reading, the texts were slightly visible initially, and, when the readers traced the letters, they appeared and disappeared as in the previous experiment. The letters were presented dynamically according to the readers’ finger movements using the software (Maruya et al., 2013)—that is, DTP by active movement (aDTP). Unlike in Experiment 1, in trace reading, the reader can control the presentation rate. A summary of characteristics of the three reading processes is shown in **Table 2**.

**Table 2:** Processing and conditions in the three reading methods.

Experiment	Experiment 1	Experiment 2	
	DTP	Silent reading (STP)	Trace reading (aDTP)
Visual processing	+	+	+
Self-paced	-	+	+
Non-limited view	-	+	-
Motor control	-	-	+ (Finger movement)

In this experiment, the optimum rate of DTP obtained in Experiment 1 was compared with the voluntary reading processes—that is, silent (STP) and trace reading (aDTP). The optimum rate in Experiment 1 was the silent reading rate of DTP. Thus, if DTP had no large influence on the optimum rate, it approximated the silent reading rate (STP) in Experiment 2. On the other hand, if DTP affected the optimum rate, it approximated the trace reading rate (aDTP).

### Method

#### Participants and Stimuli

Stimuli and participants were the same as in Experiment 1. This experiment was conducted on the same day as Experiment 1.

#### Procedure

**FIGURE 6:**
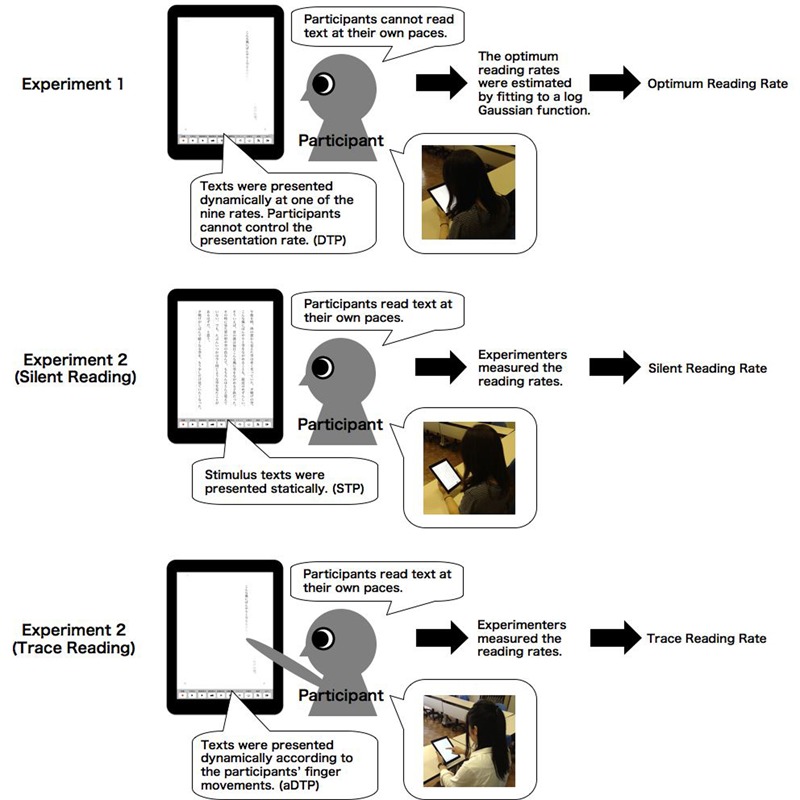
Illustration of the reading rates compared in Experiment 2.

In this experiment, silent and trace reading rates at which participants read the text at their own paces were measured. Texts were displayed at the maximum contrast using tablet computers (iPad), and the letter size, font, and colors of letters and background were the same as in Experiment 1. The experimental situation was illustrated in **Figure 6**. In silent reading, texts were presented statically, and participants read the two texts (Sunset and Snow Country) silently. The participants started reading at the experimenter’s cue. When the participants finished reading, they reported it orally. The experimenter measured the duration from the cue to the report as reading time. In trace reading, the participants read texts by tracing texts on the screen using their fingers. The letters were presented dynamically according to the readers’ finger movements. They started tracing at the experimenter’s cue, and, when they finished reading, they reported it orally. The time required for reading was measured once for each text in the two reading conditions.

### Results

**FIGURE 7:**
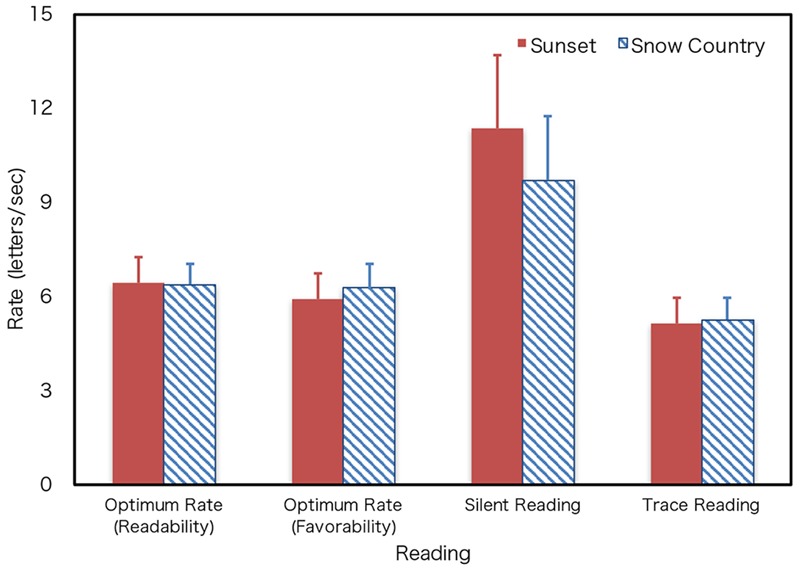
Optimum rate and reading rates. Error bars show the 95% confidence intervals.

The results demonstrate that silent reading is fast. The average silent reading rate was 11.34 letters/s, and the trace reading rate was 5.12 letters/s. **Figure 7** shows the reading rates of silent and trace reading in relation to the optimum presentation rate for readability and favorability obtained by fitting individual datasets in Experiment 1. The silent reading rate was higher than the optimum rate obtained in Experiment 1. Since one dataset (Sunset) was excluded (it was identified as an outlier by Smirnov-Grubbs test), 29 datasets (15 for Sunset and 14 for Snow Country) were used for the analyses. **Table 3** shows the results of the two-way mixed ANOVA of reading and text. The degrees of freedom were corrected using Greenhouse–Geisser correction when the Mauchly’s sphericity test was found to be significant. The main effects of the text (*F*[1,27] = 0.48, n.s.) and the interaction of text and reading were not significant (*F*[1.26, 34.25] = 1.31, n.s.). On the other hand, the main effect of reading was significant (*F*[1.26, 34.25] = 43.51, *p* < 0.0001). The Bonferroni test for multiple comparisons revealed that the rate of silent reading (STP) was significantly higher than the rate of trace reading and the optimum rates, and the rate of trace reading (aDTP) was significantly lower than that of the optimum reading rates of DTP. Though the optimum rate and trace reading rates differed significantly, the value of the difference was about 1 letters/s. Thus, it was suggested that the difference existed but was small.

**Table 3:** Results of two-way mixed ANOVA.

Optimum rate	Effect	*F*-value, *p*-value, $\eta_{p}^{2}$ -value	Multiple comparison (Bonferroni)
Readability	Reading	^∗∗∗^ *F*[1.26,34.25] = 43.51, *p* < 0.0001, $\eta_{p}^{2}$ = 0.617	Trace Reading < Optimum rate < Silent Reading
	Text	*F*[1,27] = 0.48, n.s., $\eta_{p}^{2}$ = 0.018	–
	Reading × Text	*F*[1.27,34.25] = 1.31, n.s., $\eta_{p}^{2}$ = 0.046	–
Favorability	Reading	^∗∗∗^ *F*[1.20,32.36] = 48.31, *p* < 0.0001, $\eta_{p}^{2}$ = 0.64	Trace Reading < Optimum rate < Silent Reading
	Text	*F*[1,27] = 0.234, n.s., $\eta_{p}^{2}$ = 0.01	–
	Reading × Text	*F*[1.20,32.36] = 1.78, n.s., $\eta_{p}^{2}$ = 0.06	–

*^∗∗∗^*p* < 0.0001.*

**FIGURE 8:**
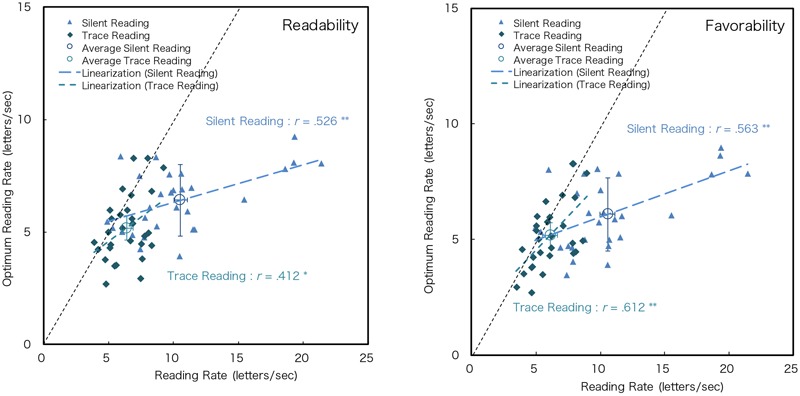
Reading rate and the optimum rate of readability and favorability. A black dotted line shows y = x. ^∗^*p* < 0.05, ^∗∗^*p* < 0.01.

**FIGURE 9:**
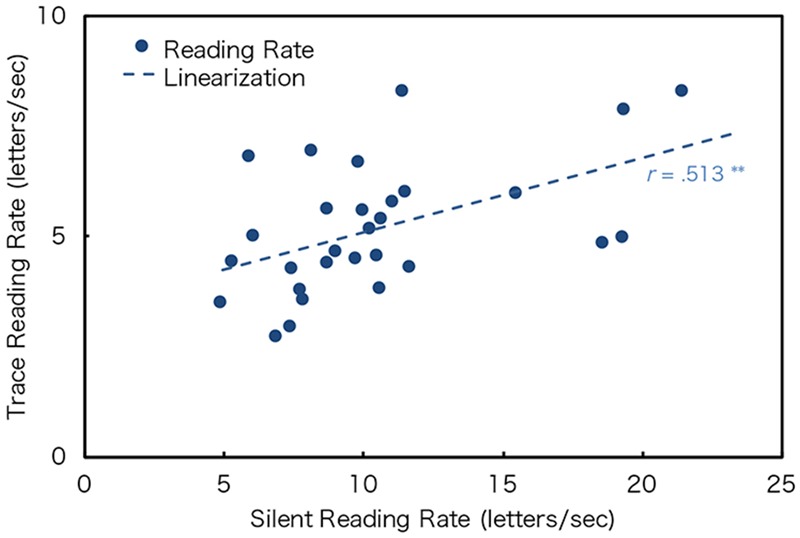
Silent reading rate and trace reading rate. ^∗∗^*p* < 0.01.

**Figure 8** shows the scatter plot of the individual datasets for the optimum rate of DTP (y-axis) to the silent (STP) and trace reading (aDTP) (x-axis) for readability and favorability. Note that the data of two text types were merged since the text factor was not significant. Pearson’s product-moment correlation coefficients of the optimum rate of DTP and silent reading for readability and favorability showed significant positive correlations, although the absolute values of reading rates differed between them (**Figure 8**). This indicates that people who can read static text fast can also read dynamically presented texts fast. Furthermore, the coefficient of the optimum rate of DTP and trace reading rates showed a significant positive correlation (**Figure 9**). There was no significant difference in reading traits for dynamically presented texts regardless of active or passive presentation.

## Discussion

The present study investigated the reading traits for dynamically presented texts. Experiment 1 demonstrated that the change of impression showed an inverted U shape and was better at around 6 letters/s. The obtained optimum rate, 6 letters/s, was similar to the average reading rate of the Japanese newsreaders (6.6 letters/s) (Mogami, 1999). The results demonstrate that the optimum rate exists in DTP and that the rate is similar to the oral reading rate of newsreaders. Unlike our prediction that readability would not decrease at lower rates, both readability and favorability decreased at lower rates. This suggests that readability and favorability are interrelated and that too-long presentation is not easy to read despite sufficient processing time.

It is known that the difficulty level of a sentence affects the memory of the text. For example, Forster (1970) found that simple one-clause sentences are recalled more correctly than complex two-clause sentences in rapid serial visual presentation. Forster and Ryder (1971) also found that semantically plausible sentences are recalled more correctly than strange sentences. As for the interaction between the difficulty level of a sentence and the speed of DTP, the reading rates of Snow Country (difficult text) at the peak values of readability and favorability were lower than that of Sunset (easy text), as shown in **Figures 3, 4**. The rating values at higher rates of Snow Country were also higher than those of Sunset, and those at lower rates of Snow Country were lower than those of Sunset. These results may demonstrate that complex texts require more reading time, but too long reading time is also undesirable. However, the significant interaction between text difficulty and DTP speed were not observed statistically. It indicates some possibilities. First, the difference of text difficulty between the texts we used is too small. Further study should use more difficult texts. Second, we did not use a comprehension test. If a comprehension test had been used in our study, participants might have perceived that they could not answer questions about Snow Country (difficult text) and noticed that they had to read the text more slowly or quickly. Third, the text difficulty might exist locally—that is, some sentences might be easy, and some might be difficult. In this case, the reading rates should be changed for each sentence according to sentence difficulty. However, only total reading rates were measured in this study. It may be necessary for the reading rates of each sentence to be measured to detect the effect of text difficulty in further study.

In addition to the optimum rate of DTP, Experiment 2 measured the silent (STP) and trace reading rates (aDTP). The three reading types differed in their processing and conditions, and all of them involved visual processing of letters (**Table 2**). Silent reading, which does not require motor control except for eye movement, was the fastest. Interestingly, the optimum rate was much lower than for silent reading, though we predicted that the optimum rate of DTP might be similar to reading at one’s own pace (silent reading). The difference was that the texts were not presented all of the time and vanished after a certain period in DTP. One possible reason for the decline in performance might be the restriction of the information of preceding letters. A reader might be able to gain text information from the preceding letters (Rayner et al., 2010; Rayner, 2014). Another reason is the limited number of displayed letters. Chujyo et al. (1993) investigated the relationship between the number of letters and the reading rate in scroll presentation. Although they reported that the optimum rate increased as the number of letters increased, the optimum rate became stable over 5 letters/frame. In Experiment 1, the number of letters increased with the presentation rate, and the number of letters presented was over 5 letters/frame at the slowest condition. Thus, it is less likely that number of letters itself played an important role in decreasing the optimum rate.

The trace reading rate was significantly low among the three reading types (**Table 3**). The finger trace movement might decrease the reading rate. In addition, it is noteworthy that the rate of trace reading shows a significant correlation with the optimum rate of dynamic text and that the difference between the trace reading rate and the optimum rate was small. It is assumed that the same factor affects the lowness of both optimum and trace reading rates. It might not be apparent that, in DTP, phonological processing occurs internally, because the text is presented sequentially, and this situation resembles acoustic processing (Potter et al., 1980; Just et al., 1982). If phonological processing occurs, it might become loaded and might cause a low optimum rate. However, it is not apparent that the slowness of dynamic text reading stemmed from the limit of the phonological process like internal speech.

We summarized the characteristics of DTP based on our findings. This study demonstrated that there is an optimum presentation rate of DTP, around 6 letters/s in our condition. This indicates that the presentation rate of dynamic text can affect the impression of reading. Interestingly, this rate is much lower than the silent reading rate (STP), though the optimum rate (DTP) was measured under the silent reading condition. The optimum DTP rate has significant correlation with the silent (STP) and trace reading rates (aDTP), and it better approximates the trace reading rate than the silent reading rate. In designing DTP, the appropriate DTP rate should be about the trace reading rate. It is assumed that the lowness of the optimum rate stems from the unique processing limit for the DTP itself. At least two possibilities exist here. First, there might be a dedicated process for dynamic text, and this process might take time. Second, translating dynamic letters to representations available in sentence processing may take time. The former is implausible because it is supposed that the common system of sentence processing is used despite the input modalities and the presentation methods. Thus, the latter possibility is more likely, but it is not evident which process takes time. This necessitates further studies. The results also demonstrated that the optimum rate was correlated with the silent (STP) and trace reading rates (aDTP). It was indicated that the readers who prefer a high reading rate in DTP, would also have a high reading speed in silent and trace reading.

Our study has some other limitations not mentioned above. First, it is unclear that our findings can be generalized because all participants and stimulus texts were Japanese. Japanese written language includes both ideograms (Chinese character) and phonograms (kana). The findings should be confirmed in other languages, especially those using the Latin alphabet, that are quite different from Japanese written language. Second, only two texts were used in this study. Our findings should be confirmed with wide range of texts. Moreover, participants read the text 27 times in Experiment 1. Thus, our findings may not be applicable to sight reading. Third, the participants in this study were relatively young. However, it is plausible that age influences the optimum rate of DTP. For example, it is predictive that the reading rate of younger people is higher than that of older people. Participants with a wide range of age classes should be included in further studies. Fourth, texts were presented with black letters and white background, and the color of stimuli was not examined in this study. Fifth, participants were not tested for their comprehension of the texts, because we focused on impression of reading. It is necessary to examine whether the optimum rate of reading impression is approximate to the optimum rate of reading comprehension.

The present study examined reading traits for dynamically presented texts. It is certain that DTP gives readers a rich reading experience not realized in traditional reading. Although the letters appear at a constant speed in our experiment, when speed and timing are changed, the temporal information of the change in text presentation might play a similar role to prosody in spoken language (Potter, 1984). In other words, such temporal features in text presentation might add information of “visual prosody” to the written language. Novel and people-friendly reading experiences in digital books, TV, or films can be achieved by utilizing DTP.

## Ethics Statement

This study was carried out in accordance with the recommendations of Provisions of Experiments, Ethics Committee of Hakodate Junior College with written informed consent from all subjects. All subjects gave written informed consent in accordance with the Declaration of Helsinki. The protocol was approved by the Ethics Committee of Hakodate Junior College.

## Author Contributions

MU, KM, and JW conceived and designed the experiments. MU and KM performed the experiments with the aid of HA at Hakodate Junior College and Osaka University. MU analyzed the data, and MU, KM, and JW wrote up the study. KM, JW, and HA contributed the software.

## Conflict of Interest Statement

The authors declare that the research was conducted in the absence of any commercial or financial relationships that could be construed as a potential conflict of interest.
